# Demographics and Yield–Per–Recruit Assessment of the Vulnerable Spiny Lobster *Palinurus elephas* in the Azores—Implications for Conservation and Fisheries Management

**DOI:** 10.3390/biology11030474

**Published:** 2022-03-19

**Authors:** Régis Santos, Ualerson Iran Peixoto, Wendell Medeiros-Leal, Rui M. Sequeira, Ana Novoa-Pabon, Mário Pinho

**Affiliations:** 1 Okeanos—UAc Instituto de Investigação em Ciências do Mar, Universidade dos Açores, Rua Prof. Dr. Frederico Machado, 4, 9900-138 Horta, Portugal; ualerson.ip.silva@uac.pt (U.I.P.); wendell.mm.silva@uac.pt (W.M.-L.); ana.mn.pabon@uac.pt (A.N.-P.); mario.rr.pinho@uac.pt (M.P.); 2 IMAR Instituto do Mar, Departamento de Oceanografia e Pescas, Universidade dos Açores, Rua Prof. Dr. Frederico Machado, 4, 9901-862 Horta, Portugal; 3 Serviço de Ambiente e Alterações Climáticas de São Jorge, Secretaria Regional do Ambiente e Alterações Climáticas, Governo dos Açores, Rua Nova—Relvinha, 9850-042 Calheta, Portugal; rmsequeira@sapo.pt

**Keywords:** crustacean, commercial species, data-limited, life-history, population structure, exploitation level, size-based method, Northeast Atlantic

## Abstract

**Simple Summary:**

The spiny lobster *Palinurus elephas* has been intensively harvested across its range and is generally considered overfished, with global landings declining sharply in the last few years. Despite its economic and ecological importance, limited information is available to perform stock assessments and make robust management decisions. Here, demographics and exploitation levels of *P. elephas* were determined from coastal areas of the Azores, and the relevance of these data for fisheries management and conservation planning was discussed.

**Abstract:**

The spiny lobster *Palinurus elephas* has been intensively harvested across its range and is generally considered overfished, with global landings declining sharply from an average of 820 t in 1960–79 to 385 t in 2000–19. Despite its economic and ecological importance, limited information is available to perform stock assessments and make robust management decisions. In this study, demographics and exploitation levels of *P. elephas* were determined from coastal areas of the Azores, and the relevance of these data for conservation planning was discussed. Carapace length varied between 39.6 and 174.3 mm, with mean sizes decreasing by depth. Males reached larger sizes and grew faster than females but were less dominant. Lifespan was 43 years for females and 60 years for males. The estimated mean length at first capture was 101.65 mm—around 58% of its asymptotic length. Fishing mortality and exploitation levels were close to the optimal values, indicating an uncertain future for Azorean populations if stock assessment and management initiatives are not focused on this species. Conservation and management strategies may benefit from these results under the ‘precautionary approach’ principle. However, up–to–date and accurate catch and fishing effort data need to be urgently collected.

## 1. Introduction

The common spiny lobster *Palinurus elephas* [1] is a temperate water species distributed in the Eastern Atlantic, from Norway to Morocco, including the Azores, Madeira and Canary archipelagos, and throughout the Mediterranean Sea [2,3]. The species occurs between the shore and 200 m depth on rocky and coralligenous bottoms, with numerous protective holes and micro-caves [4]. Adults live alone, in couples, or small groups, and, like juveniles, are predominantly active at night. However, their movements are often restricted, and their behaviour is driven by foraging and reproduction [3].

*Palinurus elephas* is one of the most abundant and accessible spiny lobsters (Family: Palinuridae) in European waters and has traditionally been a primary target for fisheries off Ireland, the United Kingdom, France, Portugal, Spain, Italy, Greece, Tunisia, and Morocco, as well as adjacent Mediterranean waters [3]. Until the 1960s and 1970s, spiny lobsters were primarily caught by traps and pots, and sometimes by diving [5,6,7]. After this period, an important change in fishing gear occurred, with the gradual introduction of trammel and tangle nets [3]. The change in fishing techniques and the increase in effort due to the fishing fleet modernization have influenced global *P. elephas* catches, which declined drastically from an average of 820 t in 1960–79 to an average of 385 t in 2000–19 [8].

Given the species’ high first-sale price (25–120 euros per kg; [9,10]), moderate mobility, and relative scarcity, *P. elephas* has been intensively harvested across its range and is generally considered overfished, although long-term catch per unit effort (CPUE) data for most fisheries are not available [4,9]. Published declines in CPUE (e.g., [3,11,12]) and widespread concern over the lack of accurate assessments of its status led to the classification of *P. elephas* by the International Union for Conservation of Nature (IUCN) as vulnerable [13].

In Portugal, *P. elephas* is fished by an artisanal fleet owning a polyvalent fishing license. Fishing occurs throughout the year, with the peak being between March and September, and catches are usually sold in local fish markets. During the last few years, landings have declined sharply from an average of 41 t in 2015–17 to 28 t in 2018–20 [14], and the species has probably disappeared from depths shallower than 30 m [3]. The decline in the fishery can partly be explained by an increase in the proportion of lobsters sold outside the legal market, in addition to the changes in the fishing operation mentioned above. The main national pieces of regulation designed explicitly for spiny lobster are the minimum landing size (95 mm carapace length) and the restriction on landing ovigerous females [15]. In addition, lobster trap fishing in Portuguese waters is permitted only from January 1 to September 30, and in the waters of the Azores sub-area of the national exclusive economic zone, fishing for female specimens is prohibited between January 1 and March 31 [16]. Only two studies about *P. elephas* have been published to date from the Portuguese coast, resulting from sampling landings in the ports of Sines in 1958 [17] and Sagres in 1993–94 and 2003 [18], both in mainland Portugal.

In the present study, the demographics and exploitation levels of *P. elephas* were determined from coastal areas of the Portuguese remote outer region of the Azores, and the relevance of these data for future conservation planning was discussed. The Azores (36° to 40° N and 24° to 32° W) is an archipelago composed of nine islands with almost no geological continental shelf [19]. Fisheries in the Azores are classified as small-scale because around 60% of the vessels are smaller than nine meters in length and target many different species [20]. This activity benefits local communities in several ways, including cultural aspects, employment, revenue generation, and food security. However, Azorean fisheries are commonly data-deficient and the status of stocks they exploit is rarely assessed [21]. Official landing statistics from the National Statistics Institute [14] for *P. elephas* show a dramatic decline from 23 t in 2016 to 3 t in 2020 in the Azores. There is also a global lack of knowledge about the basic life history and population parameters, like age, growth, and maturity, when compared to other commercial lobster species (e.g., [22,23]). This means that assessing stock health and dynamics and providing management advice is still a major focus of spiny lobster research worldwide. This study will provide primary data on the little-known *P. elephas* species and serve as a baseline to guide future research requirements, decide robust conservation actions, and define sustainable levels of exploitation and management measures.

## 2. Materials and Methods

### 2.1. Data Collection

Exploratory fishing operations occurred randomly around the Faial and São Jorge islands in the central portion of the Azores archipelago between July 2000 and February 2001. A total of 550 sets, each with 15 semi-cylindrical traps, were performed onboard traditional commercial fishing vessels. Each trap measured 67 × 46 cm in base length and 37 cm in height, and included a single-entry funnel with an inner diameter of 19 cm at the top. The bait was Atlantic chub mackerel *Scomber colias* Gmelin, 1789, and immersion time was about two days. Mean depth by set was estimated as the average between the minimum and maximum depth of the gear observed during the recovery. Catches were separated by trap and set, and for each spiny lobster, the following information was recorded: sex; total wet weight (WW; to the nearest 0.1 g); and carapace length (CL; to the nearest 0.1 mm), measured as the distance from the orbital notch inside the orbital spine to the posterior edge of the cephalothorax.

### 2.2. Data Analyses

The spiny lobster biomass was estimated using catch per unit effort (CPUE; ind. Trap^−1^). The CPUE data were then classified into depth strata delimited by a 20 m interval, ranging from 0 to 240 m. Differences in mean CPUE and CL among depth strata were determined by Welch’s heteroscedastic *F* test and Bonferroni post–hoc correction, using the ‘onewaytests’ package [24] in R [25] and assuming unequal variance between samples.

The proportion of males to females (M:F) was estimated by class (CL) and depth stratum. First, the Chi-square test was performed to evaluate if proportions diverged considerably from 1:1. Differences in mean CL between sexes were tested using the Welch’s heteroscedastic *F* test and Bonferroni post–hoc correction. Next, the relationship between CL and WW (WW = *a* × CL*^b^*) was calculated for males, females and pooled sexes. After residual analysis, the log-transformed CL and WW were used to determine the parameters *a* (intercept) and *b* (allometric coefficient) through simple linear regression (least-squares approach), using the ‘FSA’ R package [26]. For each CL–WW relationship, the *t*-test was used to verify if there was a significant difference (*p*-value < 0.05) between the isometric growth (*b* = 3) and the estimated *b* value of the equation. Afterward, the ANOVA test was used to determine if the parameters of the CL–WW relationships differed significantly between males and females.

Growth parameters were estimated for males, females and pooled sexes through the von Bertalanffy growth function (VBGF) [27] using monthly CL-frequency data (2 mm class interval). The original VBGF model [27] was modified as follows to remove theoretical age at length zero (*t*_0_):

*L_t_* = *L_∞_* (1 − e^−*k*(*t*)^)

where *L_t_* is the length (cm) at age *t* (year), *L_∞_* is the asymptotic length (cm), and *k* is the growth coefficient (year^−1^). The mean ± 0.95 confidence interval values of the asymptotic length (*L_∞_*), growth coefficient (*k*), and growth performance index (ϕ′) were computed by electronic length–frequency analysis using a bootstrapped method with a genetic algorithm (ELEFAN_GA_boot; [28]) within the ‘TropFishR’ [29,30] and ‘fishboot’ [28,31] packages. Bootstrap estimates were based on 1000 resamples. The lifespan (*t_max_*) was calculated as *t_max_* = 3/*k* [32].

Total mortality rate (*Z*; year^−1^) was calculated based on the linearized length–converted catch curve [33] within the ‘TropFishR’ package [29,30]. Natural mortality (*M*; year^−1^) was estimated as the mean value of *M* computed using different empirical equations [34,35,36,37,38,39,40,41,42,43,44,45]. Fishing mortality (*F*; year^−1^) was determined from the relationship *F* = *Z* − *M*. The current exploitation rate (*E*; year^−1^) was obtained as *E = F*/(*F* + *M*) [46].

The CL at first capture where 50% of the individuals are retained by the gear (*L_c_*) was determined using a logit function on the capture probability. Then, the relative yield–per–recruit (Y′/R) and relative biomass–per–recruit (B′/R) analyses were performed according to the Beverton–Holt method [47] to determine the exploitation level using the estimated growth and mortality parameters within the ‘TropFishR’ package [29,30]. This method generates different catch curves based on different fishing mortalities. It calculates the exploitation rate for the maximum yield (*E_max_*), with *E*_50_ denoting the exploitation rate under which the stock has been reduced to 50% of its virgin biomass and *E*_10_ the optimal exploitation rate at which the marginal increase of Y′/R is 1/10 of its value at *E* = 0.

Due to poor data adjustment, no sex-specific mortality and exploitation rates, or yield-per-recruit assessments were performed.

## 3. Results

### 3.1. Abundance and Size Structure

**Figure 1:**
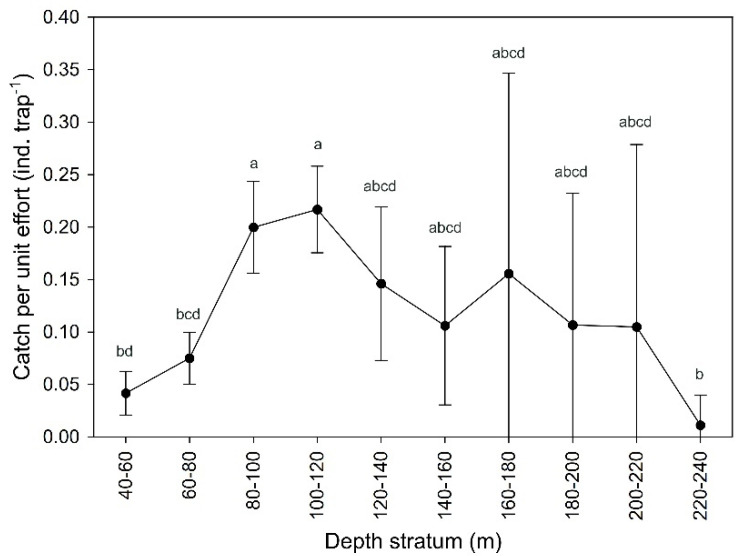
Mean ± 0.95 confidence interval catches per unit effort (CPUE, ind. trap^−1^) by depth stratum of *Palinurus elephas* from the Azores for the period 2000–01. Different letters indicate significant differences between groups (Bonferroni post–hoc correction, *p*-value < 0.05).

Failure to collect fishing information (e.g., equipment problems, weather conditions) invalidated 34 fishing sets (6% of the total). From the remaining 516 sets, 1128 individuals were caught between 40 and 222 m depth. Significant statistical differences were found in the mean CPUE (ind. trap^−1^) by depth stratum (Welch’s test, F = 12.21, *p* < 0.001). Abundances were higher between 80 and 120 m than between 40 and 80 m and 220 and 240 m (Figure 1; Table S1).

**Figure 2:**
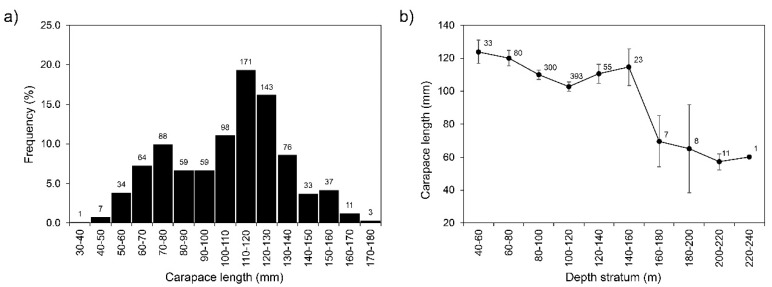
Length-frequency distribution (**a**); and mean ± 0.95 confidence interval carapace length (mm) by depth stratum (**b**) of *Palinurus elephas* from the Azores sampled during the 2000–01 period. Numbers inside the graph represent the number of individuals (*n*).

Carapace length (CL) varied between 39.6 and 174.3 mm (mean ± standard deviation: 107.0 ± 27.1 mm). Most captured individuals were in the 110–120 mm CL-class (*n* = 171; Figure 2a). Mean CL decreased significantly by depth (Welch’s test, F = 70.53, *p* < 0.001) with larger individuals at 40–60 m and smaller ones at deeper (200–220 m) strata (Figure 2b; Table S2).

### 3.2. Sex

**Figure 3:**
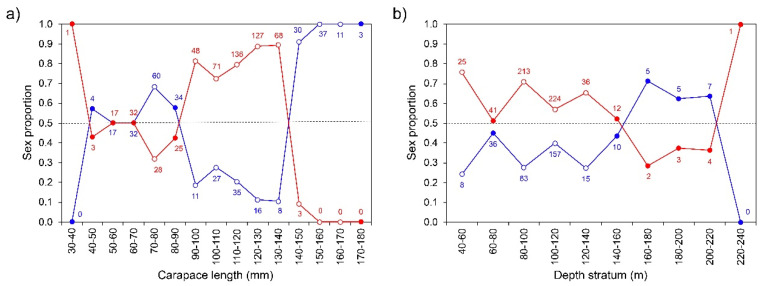
Proportion of males (in blue) and females (in red) of *Palinurus elephas* by size class (**a**); and depth stratum (**b**) in the Azorean region during the period 2000–01. Numbers inside the graph represent the number of individuals (*n*). Open circles denote statistically significant differences (*p*-value < 0.05) between sexes.

The overall sex ratio (0.61:1) showed a statistically significant (Chi–square test, χ^2^ = 64.09, *p* < 0.001) predominance of females. Carapace length ranged from 39.6 to 147.1 mm (mean ± standard deviation: 108.3 ± 21.9 mm) in females and 44.5 to 174.3 mm (104.7 ± 34.2 mm) in males. Difference between sexes in the mean CL was not statistically significant (Welch’s test, F = 2.87, *p* = 0.091). However, individuals measuring 90 to 140 mm CL were predominantly females (χ^2^ > 19.76, *p* < 0.001; Figure 3a; Table S3), and individuals larger than 140 mm were mostly males (χ^2^ > 11.00, *p* < 0.001; Figure 3a; Table S3). At shallower depth strata (up to 140 m), females were significantly more abundant than males (χ^2^ > 8.31, *p* < 0.004; Figure 3b; Table S3).

### 3.3. Length–Weight Relationships

The linear regression models indicated that the species grows faster than it gains weight (WW), indicating that the species grows allometrically (*b* < 3). Table 1 summarizes the parameters of the CL–WW relationships. ANOVA findings for the differences in CL–WW relationships between males and females suggested that the interaction terms were not significant (F = 3.12, *p* = 0.078; Table S4). Thus, there is insufficient evidence to conclude that the slopes of the CL–WW relationship differed between the sexes. The *p*-value for the indicator variable suggested a difference in intercepts between the two sexes (F = 20,435.03, *p* < 0.001; Table S4). Because the two sexes had statistically equal slopes but different intercepts, there was a constant difference between the log-transformed weights of individuals from the two sexes regardless of their log-transformed lengths. The confidence intervals showed that females were between 0.060 and 0.107 heavier, on the log-scale, than males, regardless of the length of the individual (Table S4; Figure S1).

### 3.4. Growth, Life Span, and Mortality

**Figure 4:**
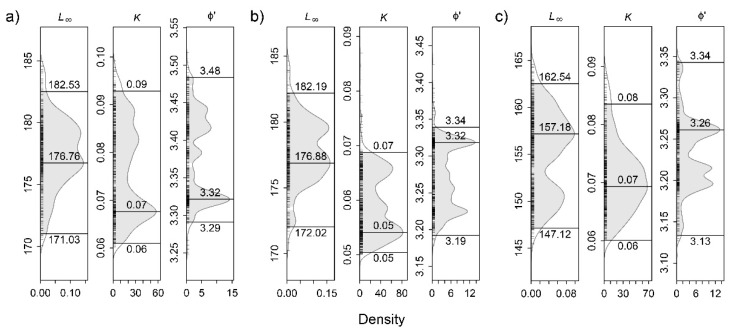
Estimates of asymptotic length (*L_∞_;* cm), growth rate coefficient (*k*; year^−1^), and growth performance index (ϕ′) with 0.95 confidence intervals for all *Palinurus elephas* sampled in the Azores during the 2000–01 period (**a**); and for males (**b**); and females (**c**), separately.

The estimated growth parameters (asymptotic length—*L_∞_*, growth coefficient—*k*, and growth performance index—ϕ′) and their 0.95 confidence intervals are shown in Figure 4. The best fitted parameters obtained from CL–frequency data for the period 2000–2001 were *L_∞_* = 176.76 mm CL; *k* = 0.07 year^−1^; and ϕ′ = 3.32 for pooled sexes, *L_∞_* = 176.88 mm CL, *k* = 0.05 year^−1^ and ϕ′ = 3.32 for males, and *L_∞_* = 157.18 mm CL, *k* = 0.07 year^−1^ and ϕ′ = 3.26 for females.

Lifespan (*t_max_*) was estimated to be 42.86 years in pooled sexes and females and 60.00 years in males.

**Figure 5:**
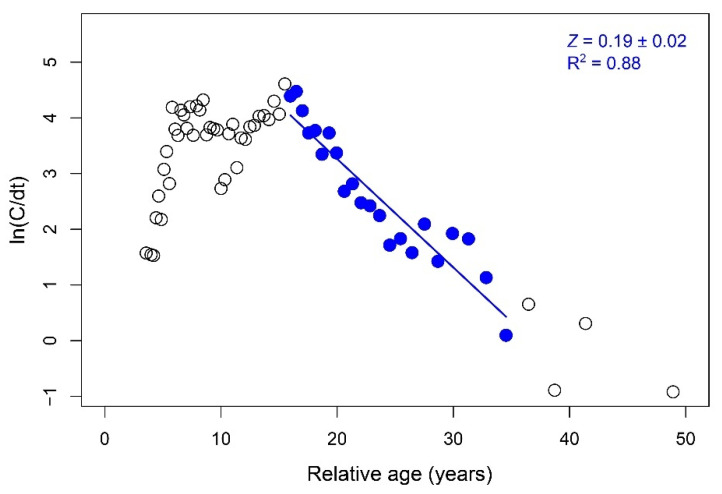
Estimates of total mortality rate (*Z*; mean ± 0.95 confidence interval) for *Palinurus elephas* in the Azores from the linearized length–converted catch curve method.

Total mortality (*Z*), natural mortality (*M*), and fishing mortality (*F*) for the period 2000–01 were estimated at 0.19 year^−1^, 0.13 year^−1^, and 0.06 year^−1^, respectively. Details on the estimated values are shown in Figure 5 and Table 2.

### 3.5. Exploitation

**Figure 6:**
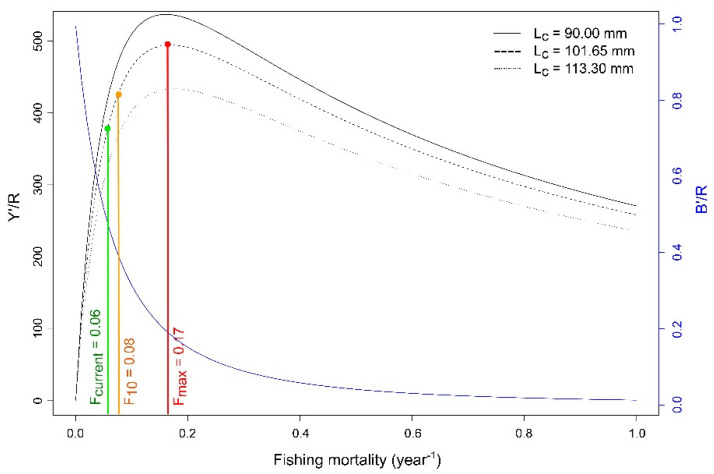
Relative yield–per–recruit (Y′/R; g year^−1^) and biomass–per–recruit (B′/R; g) based on the model of Beverton and Holt (1957) for *Palinurus elephas* sampled in the Azores during the 2000–01 period. *F_current_* (year^−1^): fishing mortality (*F*) found in the present study; *F*_10_ (year^−1^): fishing mortality rate corresponding to 10% of the slope of the Y′/R curve at the origin*; F_max_* (year^−1^): fishing mortality rate which produces the maximum Y′/R. *L_c_*: carapace length at first capture (mm).

The estimated mean length at first capture (*L_c_*) for *P. elephas* was 101.65 mm CL (i.e., 12.22 years old)—around 58% of its asymptotic length (Table 3). The current *F* was quite near to the optimum level of fishing mortality that corresponds to 10% of the slope of the yield–per–recruit (Y′/R) curve at the origin (i.e., *F*_10_; Figure 6). The current exploitation rate (*E* = 0.33 year^−1^; Table 2) was smaller than the exploitation rate for the maximum yield (*E_max_* = 0.56 year^−1^), greater than the exploitation rate under which the stock has been reduced to 50% of its virgin biomass (*E*_50_ = 0.27 year^−1^), and close to the optimal exploitation level (*E*_10_ = 0.38 year^−1^). A reduction in *L_c_* to 90 mm CL (i.e., 10.16 years old) implies an increase in *F*_10_ from 0.08 to 0.12 year^−1^; however, the values of *E*_10_ and *E_max_* become relatively close to each other (Table 3).

## 4. Discussion

Aquatic living resources can renew themselves as populations by increasing their size and weight, and by reproducing. In an equilibrium population, these additive processes of growth and reproduction equal the loss process of natural and fishing mortality [50]. However, in an exploited population, fisheries management should ensure that fishing mortality does not exceed the level that the population can tolerate, in addition to natural mortality, without causing excessive harm or compromising the population’s sustainability and productivity [51]. The definition of this level and pattern of fishing mortality is very dependent on how much information there is about the target species’ abundance and population dynamics that can be used for stock assessment.

Despite its economic and ecological importance, limited information about *Palinurus elephas* is available to make robust management decisions where it occurs and is commercially exploited. Official landings obtained from FAO Global Capture Production statistics [8] for the Atlantic fisheries reveal a sharp decline from 198 t in 1991 to 20 t in 2019. For the Mediterranean, *P. elephas* landings reached 1000 t in the 1960s and 1970s, declined to 180 t in 2000, then recovered to 356 t in 2019. However, a reconstruction of Corsican landings from 1950 to 2008 indicated that 16 times the amount recorded to the FAO actually landed, but a decline in captures was still evident in recent years [52]. Even though they are under-reported, these consistent declines appear to have fishing mortality as the primary cause [9].

For the Azores archipelago, the results presented herein represent the first estimates ever obtained of the demographics and exploitation levels of *P. elephas* in the region. The findings extended the species’ previously described depth distribution range from 200 m to 222 m depth [3] and identified a maximum abundance between 80 and 120 m. In the western Mediterranean Sea, adult spiny lobsters are mainly found at 50–100 m depth [4]. The maximum observed carapace length of the species was 200 mm [53], with a review study observing that Atlantic *P. elephas* attains larger modal and maximum sizes than the Mediterranean populations [9]. Individuals of *P. elephas* measuring 174 mm observed in the present study were, therefore, smaller than the maximum reported size of the species and the maximum recorded length for mainland Portugal (i.e., 193 mm; [17]). The spatial distribution and maximum size of spiny lobsters in an exploited population, however, is dependent on the level and pattern of exploitation, and observed values should be interpreted cautiously since they may also be influenced by many parameters such as sample size, sampling technique, habitat, season, and depth [3].

Mark-recapture studies performed in the Atlantic and the Mediterranean demonstrated minimal adult movement, with most individuals moving less than 10 km [54,55,56,57,58]. On the other hand, seasonal migration for moulting and reproduction is known to occur, and episodic migration for escaping from unfavourable environmental conditions [59]. In the Atlantic, *P. elephas* migrates onshore during the pre-reproductive spring season and then offshore during the post-reproductive autumn season [60,61]. In addition, the settlement depth (10–15 m) is known to be much shallower than the depths at which adults are typically found, i.e., depths greater than 40 m [62]. Therefore, the smaller-deeper distribution pattern observed in this study (Figure 2) indicates that after settlement, juveniles (>30 mm CL) migrate to deeper regions than those occupied by adults (>75 mm CL, considering the smaller reported size that they reach physiological and functional maturity; [9]). Such depth segregation presumably limits intraspecific competition for available space and food supplies, as inferred for other crustacean species (e.g., [63,64,65,66]).

The observed pattern of increased female abundance in shallower waters was aligned with the onshore–offshore movements of *P. elephas* and is also seen in the Atlantic [60,61,67] and temperate spiny lobster populations [59]. Sexual dimorphism in CL with males in the large-size group and females in the medium-size group is common in this spiny lobster species [3,68]. According to the growth data analysis, male spiny lobsters had a faster growth rate than female spiny lobsters (Figure S2). However, this difference seems more pronounced after sexual maturity is reached, when the females start investing more energy in reproduction than in body growth [69,70,71]. Females growing slower than males after sexual maturity is also a growth pattern observed in adults of its congeners, *P. gilchristi* and *P. delagoae* [72,73].

The theoretical maximum lengths (*L_∞_*) and growth coefficients (*k*) of the von Bertalanffy growth function estimated in this study are the first for the Atlantic. Overall, *P. elephas* attains a larger *L_∞_* and lower *k* in the Azores than in the Mediterranean Sea (Table 4). These differences may be attributed to several factors such as exploitation level, food availability, environmental characteristics (e.g., temperature, dissolved oxygen), and population density [3,69,74,75,76]. In the Mediterranean, for example, the substantial effort increase through the use of trammel-nets has been pointed out as being responsible for the reduction in the number of large individuals in the population [3]. Regarding growth rates, studies on lobster species suggest that growth is reduced where colder seasonal seawater temperatures are observed, such as in the northern areas of the Atlantic [77]. Additionally, aggressive social interactions in dense populations may result in growth rate decreases, independent of food availability due to increasing competitiveness [69]. Further research would therefore help clarify the reasons for the differences in growth rates between Atlantic and Mediterranean regions.

The length–weight relationship parameters for males and females were within the range described for *P. elephas* fished in the Atlantic [18,60,61,81] and Mediterranean [53,82], in which the main characteristic is the negative allometry (*b* < 3). However, for an equal CL, females had a greater WW than males. This morphometric characteristic is associated with the size of the abdomen, which in females is larger due to its reproductive role as an egg attachment zone [82,83]. These sex differences support calculating growth curves for each sex separately. Generally, stock assessment models require a single set of population characteristics since successful management strategies cannot always be applied differently to males and females, because harvesting does not discriminate between sexes. Nevertheless, besides the differences in growth, the discrepancy in the observed sex ratio, indicating a lower catchability in traps of males than females, may warrant an adjustment in management regulations to reduce effort, particularly on the target component that is especially more susceptible to overexploitation. Because the dataset analysed in this study did not allow the progress of the assessment in this direction, and because it is impossible to determine whether reduced catchability is related to sex or size, since the largest individuals in the population are males, future studies should focus on these aspects.

Besides its large size, slow growth, and long lifespan, *P. elephas* showed low natural mortality (*M*), making it particularly vulnerable to overexploitation. The resulting estimates of *M* (0.13 year^−1^) can be compared with those estimated in previous studies on other Atlantic [5] and Mediterranean populations [55,78,79] through direct or indirect methods (Table 4). Direct exploitation of *P. elephas* has occurred in several countries throughout its range, using a variety of gears (primarily traps, pots, trammels, and tangle nets) and effort levels, and the effects of such fisheries on population status have been observed through the declining trend in short-term catch-per-unit-effort [3,11,12]. Lower levels of fishing mortality have been observed in the Azores (Table 4) compared to other regions of the Atlantic [5] and Mediterranean [55,78]. On the other hand, current fishing mortality and exploitation levels observed in this study were close to the optimal values (i.e., *F*_10_ and *E*_10_), and current exploitation levels were above the *E*_50_ value, indicating an unclear future for Azorean populations if stock assessment and management initiatives are not focused on this species.

The estimated mean carapace length at first capture (*L_c_*) was 102 mm, indicating that adults represented a large proportion of the catches. Furthermore, the size at 50% maturity of *P. elephas* has been estimated to be between 82- and 110-mm CL in Atlantic waters [18,61], suggesting that mature adults and spawning individuals may be being exploited in the Azores. However, due to the lack of information on the reproductive stages of the spiny lobsters captured, this cannot be substantiated but is noted as a reason for concern and a potential subject for further investigation. Since 2001, for example, a minimum legal size (MLS) of 95 mm CL has been in place [15], but has been proven to be inadequate to stimulate an increase in egg production on mainland Portugal, since fewer than 20% of the 95 mm CL females are mature [18]. Whereas in the Mediterranean, the MLS of 90 mm CL has been shown to protect just approximately 1% of potential population egg production [84].

## 5. Conclusions

Future conservation and management strategies for *P. elephas* may benefit from the results presented in this study under the ‘precautionary approach’ principle [85]. However, more than two decades after the data analysed here were collected, no statistics on the current condition of the spiny lobster stock off the Azorean coast are available. Official commercial landings of *P. elephas* have consistently declined in recent years in the Azores [10], and it is reasonable to assume that the exploitation status of this stock has also worsened, despite the adopted management measures as MLS [15], closed period [86], prohibition of trammels and tangle nets [87], and technical specificities for traps [88,89]. However, to confirm this, up-to-date and accurate catch and fishing effort data need to be urgently collected, as well as the data needed to calculate new population parameter estimates, including those related to reproduction. A continuous monitoring programme of abundance and demographic trends is thus strongly suggested to enable the development of science-based management strategies that support sustainable fisheries of *P. elephas*.

## Figures and Tables

**Table 1:** Parameters of CL–WW relationship (*W* = *a* CL*^b^*) for males, females, and pooled sexes of *Palinurus elephas* caught during the period 2000–01. *a*: intercept; *b*: allometric coefficient; SE: standard error; *p*: *t*-test *p*-value; R^2^: determination coefficient; *n*: number of individuals.

Sex	*n*	*a*	*b*	SE (*a*)	SE (*b*)	R^2^	Departure from *b* = 3
Males	196	0.002	2.839	0.124	0.027	0.983	t = −5.939; *p* < 0.001
Females	215	0.003	2.769	0.132	0.029	0.977	t = −7.987; *p* < 0.001
Pooled	411	0.002	2.812	0.095	0.021	0.978	t = −9.018; *p* < 0.001

**Table 2:** Natural mortality, fishing mortality and exploitation rate estimated for *Palinurus elephas* using empirical equations based on the approximate maximum age (*t_max_*); asymptotic length (*L_∞_*); and growth coefficient (*k*). *M*: natural mortality; *T*: mean annual water temperature of the region (*T* = 18 °C; [19,48,49]). The *L_∞_* and *k* were derived from the CL-frequency data collected for the period 2000–01 in the Azores.

Parameter	Estimate	Empirical Formula	Reference
Life span (*t_max_*; years)	42.86	*t_max_* = 3/*k*	[32]
Natural mortality (*M*; year^−1^)	0.12	*M* = 5/*t_max_*	[34]
	0.07	*M* = 2.996/*t_max_*	[35]
	0.06	*M* = 2.5/*t_max_*	[38]
	0.18	M = 3 *k*/(*e* ^0.38 *tmax* × *k*^ − 1)	[39]
	0.45	*M* = exp(−0.0066 − 0.279 × log(*L_∞_*) + 0.6543 × log(*k*) + 0.4634 × log(*T*))	[40]
	0.07	*M* = 3/*t_max_*	[41]
	0.11	*M* = 4.6/*t_max_*	[42]
	0.15	M = 1.0661 × *L_∞_*^−0.1172^ × *k* ^0.5092^	[37]
	0.04	*M* = −0.1778 + 3.1687 *k*	[43]
	0.11	*M* = 1.6 *k*	[44]
	0.11	*M* = 1.5 *k*	[44]
	0.10	*M* = 1.4 *k*	[45]
	0.10	*M* = 4.22/*t_max_*	[36]
	0.13	Average *M* value	
Fishing mortality (*F*; year^−1^)	0.06	*F* = *Z* − *M*	
Exploitation rate (*E*; year^−1^)	0.33	*E* = *F*/(F + M)	[46]

**Table 3:** Estimates of carapace length at first capture (*L_c_*; mm); age at first capture (*t_c_*; years); fishing mortality rate corresponding to 10% of the slope of the Y′/R curve at the origin (*F*_10_; year^−1^); fishing mortality rate which produces the maximum Y′/R (*F_max_*; year^−1^); exploitation rate under which the stock has been reduced to 50% of its virgin biomass (*E*_50_; year^−1^); the optimal exploitation rate at which the marginal increase of Y′/R is 1/10 of its value at E = 0 (*E*_10_; year^−1^); and maximum exploitation rate which gives the maximum Y′/R (*E_max_*; year^−1^) estimated by Beverton–Holt method [47] for *Palinurus elephas*.

*L_c_*	*t_c_*	*F*_10_	*F_max_*	*E*_50_	*E*_10_	*E_max_*
90.00	10.16	0.12	0.16	0.27	0.48	0.55
101.65	12.22	0.08	0.17	0.27	0.38	0.56
113.30	14.63	0.05	0.18	0.27	0.27	0.58

**Table 4:** Summary of growth and mortality studies of *Palinurus elephas*. MR: mark–recapture; LCC: length–converted catch curve; LFD: length–frequency analysis; TA: theoretical arguments; *n*: number of individuals; CL: carapace length; P: pooled sexes; M: males; F: females; *L_∞_*: asymptotic length (cm); *k*: growth coefficient (year^−1^); *t*_0_: theoretical age at length zero; ϕ′: growth performance index; *Z*: total mortality rate (year^−1^); *M*: natural mortality rate (year^−1^); *F*: fishing mortality rate (year^−1^).

Source	Area	Method	*n*	Size	Sex	Growth Parameter				Mortality Rate		
						*L_∞_*	*k*	*t*_0_	ϕ′	*Z*	*M*	*F*
Mediterranean Sea												
[55]	Corsica	MR, TA	61	CL	M	166.0	0.15	−0.35	3.62	0.30–0.52	0.15–0.30	0.15–0.22
		MR, TA	50	CL	F	135.9	0.19	−0.34	3.54	0.23–0.42	0.15–0.30	0.08–0.12
[69]	CW Mediterranean Sea	MR	146	CL	M	167.9	0.13	−0.40	3.56			
		MR	146	CL	M	167.0	0.13	−0.40	3.56			
		MR	102	CL	F	120.2	0.21	−0.35	3.48			
		MR	102	CL	F	125.0	0.19	−0.37	3.47			
[78]	CW Mediterranean Sea	MR	147	CL	M	189.0	0.10		3.50			
		MR	107	CL	F	117.0	0.16		3.30			
		MR	254	CL	P					0.70	0.27	0.43
[79]	W Mediterranean Sea	MR	270	CL	M						0.20	
		MR	296	CL	F						0.16	
[71]	Tunisia	LFD	-	CL	M	201.6	0.16	−0.27	3.81			
		LFD	-	CL	F	155.4	0.22	−0.25	3.73			
[80]	W Mediterranean Sea	LCC	-	CL	M					0.19–0.80		
		LCC	-	CL	F					0.17–0.57		
Atlantic Ocean												
[5]	Cornwall	MR	286	CL	P					0.23	0.11	0.12
This study	Azores	LFD	325	CL	M	176.9	0.05		3.32			
		LFD	559	CL	F	157.2	0.07		3.26			
		LFD, LCC	884	CL	P	176.8	0.07		3.32	0.19	0.13	0.06

## Data Availability

The data underlying this article will be shared upon reasonable request to the corresponding author.

## Supplementary Materials

The following supporting information can be downloaded at: https://www.mdpi.com/article/10.3390/biology11030474/s1, Table S1. Results of Bonferroni post–hoc test for comparing mean catch per unit effort (ind. trap^−1^) of *Palinurus elephas* between depth strata. Significance level was set at 0.05, Table S2. Results of Bonferroni post–hoc test for comparing mean carapace lengths of *Palinurus elephas* between depth strata. Significance level was set at 0.05, Table S3. Results of Chi–square test for comparing sex ratio (M:F) of *Palinurus elephas* between size classes and depth strata. Significance level was set at 0.05. CL: carapace length, Table S4. Summary of ANOVA statistics to determine differences in length–weight relationships between sexes. WW: wet weight; CL: carapace length; fsex: sex as a group factor variable; M: males, Figure S1. Plots of the log-transformed length–weight relationships illustrating observed intercept differences between males (M) and females (F) of *Palinurus elephas* from the Azores. See Table S4 for statistic results, Figure S2. Von Bertalanffy growth curve and individual growth increments for males (a) and females (b) of *Palinurus elephas* from the Azores. Red dashed lines represent 0.95 confidence intervals.
