# Supplementary material for: Demographics and Yield–Per–Recruit Assessment of the Vulnerable Spiny Lobster Palinurus elephas in the Azores—Implications for Conservation and Fisheries Management
Source: Biology (Basel). 2022 Mar 19;11(3):474. doi: 10.3390/biology11030474 (PMC8945453; doi:10.3390/biology11030474)
Supplement: Supplementary file 1 [file biology-11-00474-s001.zip › biology-1632591-supplementary.pdf]

biology

MDPI

Article

Demographics and Yield–Per–Recruit Assessment of the Vulnerable Spiny Lobster *Palinurus Elephas* in the Azores—Implications for Conservation and Fisheries Management

Régis Santos [1,2,*], Ualerson Iran Peixoto [1,2], Wendell Medeiros-Leal [1,2], Rui M. Sequeira [3], Ana Novoa-Pabon [1] and Mário Pinho [1,2]

[1] Okeanos—UAc Instituto de Investigação em Ciências do Mar, Universidade dos Açores, Rua Prof. Dr. Frederico Machado, 4, 9900-138 Horta, Portugal; ualerson.ip.silva@uac.pt (U.I.P.); wendell.mm.silva@uac.pt (W.M.-L.); ana.mn.pabon@uac.pt (A.N.-P.); mario.rr.pinho@uac.pt (M.P.)
[2] IMAR Instituto do Mar, Departamento de Oceanografia e Pescas, Universidade dos Açores, Rua Prof. Dr. Frederico Machado, 4, 9901-862 Horta, Portugal
[3] Serviço de Ambiente e Alterações Climáticas de São Jorge, Secretaria Regional do Ambiente e Alterações Climáticas, Governo dos Açores, Rua Nova—Relvinha, 9850-042 Calheta, Portugal; rmsequeira@sapo.pt
* Correspondence: regisvinicius@gmail.com; Tel.: +351–292200400

Supplementary Materials

Table S1. Results of Bonferroni post–hoc test for comparing mean catch per unit effort (ind. trap^{-1}) of *Palinurus elephas* between depth strata. Significance level was set at 0.05.
Table S2. Results of Bonferroni post–hoc test for comparing mean carapace lengths of *Palinurus elephas* between depth strata. Significance level was set at 0.05.
Table S3. Results of Chi–square test for comparing sex ratio (M:F) of *Palinurus elephas* between size classes and depth strata. Significance level was set at 0.05. CL: carapace length.
Table S4. Summary of ANOVA statistics to determine differences in length–weight relationships between sexes. WW: wet weight; CL: carapace length; fsex: sex as a group factor variable; M: males.
Figure S1. Plots of the log-transformed length–weight relationships illustrating observed intercept differences between males (M) and females (F) of *Palinurus elephas* from the Azores. See Table S4 for statistic results.
Figure S2. Von Bertalanffy growth curve and individual growth increments for males (a) and females (b) of *Palinurus elephas* from the Azores. Red dashed lines represent 0.95 confidence intervals.

Table S1. Results of Bonferroni post–hoc test for comparing mean catch per unit effort (ind. trap^{-1}) of *Palinurus elephas* between depth strata. Significance level was set at 0.05.

Level (a)	Level (b)	*p*-Value	No Difference
100–120	120–140	1.000	Not reject
100–120	140–160	0.539	Not reject
100–120	160–180	1.000	Not reject
100–120	180–200	1.000	Not reject
100–120	200–220	1.000	Not reject
100–120	220–240	< 0.001	Reject
100–120	40–60	< 0.001	Reject
100–120	60–80	< 0.001	Reject
100–120	80–100	1.000	Not reject
120–140	140–160	1.000	Not reject
120–140	160–180	1.000	Not reject
120–140	180–200	1.000	Not reject
120–140	200–220	1.000	Not reject
120–140	220–240	0.054	Not reject
120–140	40–60	0.395	Not reject
120–140	60–80	1.000	Not reject
120–140	80–100	1.000	Not reject
140–160	160–180	1.000	Not reject
140–160	180–200	1.000	Not reject
140–160	200–220	1.000	Not reject
140–160	220–240	0.911	Not reject
140–160	40–60	1.000	Not reject
140–160	60–80	1.000	Not reject
140–160	80–100	1.000	Not reject
160–180	180–200	1.000	Not reject
160–180	200–220	1.000	Not reject
160–180	220–240	1.000	Not reject
160–180	40–60	1.000	Not reject
160–180	60–80	1.000	Not reject
160–180	80–100	1.000	Not reject
180–200	200–220	1.000	Not reject
180–200	220–240	1.000	Not reject
180–200	40–60	1.000	Not reject
180–200	60–80	1.000	Not reject
180–200	80–100	1.000	Not reject
200–220	220–240	1.000	Not reject
200–220	40–60	1.000	Not reject
200–220	60–80	1.000	Not reject
200–220	80–100	1.000	Not reject
200–220	40–60	1.000	Not reject
220–240	60–80	0.038	Reject
220–240	80–100	< 0.001	Reject
40–60	60–80	1.000	Not reject
40–60	80–100	< 0.001	Reject
60–80	80–100	< 0.001	Reject

Table S2. Results of Bonferroni post–hoc test for comparing mean carapace lengths of *Palinurus elephas* between depth strata. Significance level was set at 0.05.

Level (a)	Level (b)	*p*-Value	No Difference
100–120	120–140	0.636	Not reject
100–120	140–160	1.000	Not reject
100–120	160–180	0.060	Not reject
100–120	180–200	0.445	Not reject
100–120	200–220	< 0.001	Reject
100–120	40–60	< 0.001	Reject
100–120	60–80	< 0.001	Reject
100–120	80–100	0.013	Reject
120–140	140–160	1.000	Not reject
120–140	160–180	0.010	Reject
120–140	180–200	0.165	Not reject
120–140	200–220	< 0.001	Reject
120–140	40–60	0.130	Not reject
120–140	60–80	0.461	Not reject
120–140	80–100	1.000	Not reject
140–160	160–180	0.002	Reject
140–160	180–200	0.089	Not reject
140–160	200–220	< 0.001	Reject
140–160	40–60	1.000	Not reject
140–160	60–80	1.000	Not reject
140–160	80–100	1.000	Not reject
160–180	180–200	1.000	Not reject
160–180	200–220	1.000	Not reject
160–180	40–60	0.001	Reject
160–180	60–80	0.003	Reject
160–180	80–100	0.020	Reject
180–200	200–220	1.000	Not reject
180–200	40–60	0.034	Reject
180–200	60–80	0.057	Not reject
180–200	80–100	0.187	Not reject
200–220	40–60	< 0.001	Reject
200–220	60–80	< 0.001	Reject
200–220	80–100	< 0.001	Reject
40–60	60–80	1.000	Not reject
40–60	80–100	0.016	Reject
60–80	80–100	0.016	Reject

Table S3. Results of Chi–square test for comparing sex ratio (M:F) of *Palinurus elephas* between size classes and depth strata. Significance level was set at 0.05. CL: carapace length.

CL (mm)	Sex Ratio	Chi–Square (χ^2)	*p*–Value
30–40	0:1	1.00	0.317
40–50	1.33:1	0.14	0.705
50–60	1:1	0.00	1.000
60–70	1:1	0.00	1.000
70–80	2.14:1	11.64	0.001
80–90	1.36:1	1.37	0.241
90–100	0.23:1	23.20	< 0.001
100–110	0.38:1	19.76	< 0.001
110–120	0.26:1	59.65	< 0.001
120–130	0.13:1	86.16	< 0.001
130–140	0.12:1	47.37	< 0.001
140–150	10:1	22.09	< 0.001
150–160	1:0	37.00	< 0.001
160–170	1:0	11.00	0.001
170–180	1:0	3.00	0.083

Depth Stratum (m)	Sex Ratio	Chi–Square (χ^2)	*p*–Value
40–60	0.32:1	8.76	0.003
60–80	0.88:1	0.43	0.514
80–100	0.39:1	56.39	< 0.001
100–120	0.7:1	11.79	0.001
120–140	0.42:1	8.31	0.004
140–160	0.83:1	0.22	0.641
160–180	2.5:1	1.29	0.257
180–200	1.67:1	0.50	0.480
200–220	1.75:1	0.82	0.366
220–240	0:1	1.00	0.317

Table S4. Summary of ANOVA statistics to determine differences in length–weight relationships between sexes. WW: wet weight; CL: carapace length; fsex: sex as a group factor variable; M: males.

**Analysis of Variance Table**					
Response: logWW					
	**d.f.**	**Sum Sq**	**Mean Sq**	**F value**	***p*–value**
logCL	1	300.128	300.128	20435.03	<0.001
fsex	1	0.711	0.711	48.4233	<0.001
logCL:fsex	1	0.046	0.046	3.1209	0.078
Residuals	407	5.978	0.015		
**Confidence interval**					
	**2.5%**	**97.5%**			
(Intercept)	−6.16916	−5.81391			
logCL	2.771077	2.848631			
fsexM	−0.10689	−0.0597			

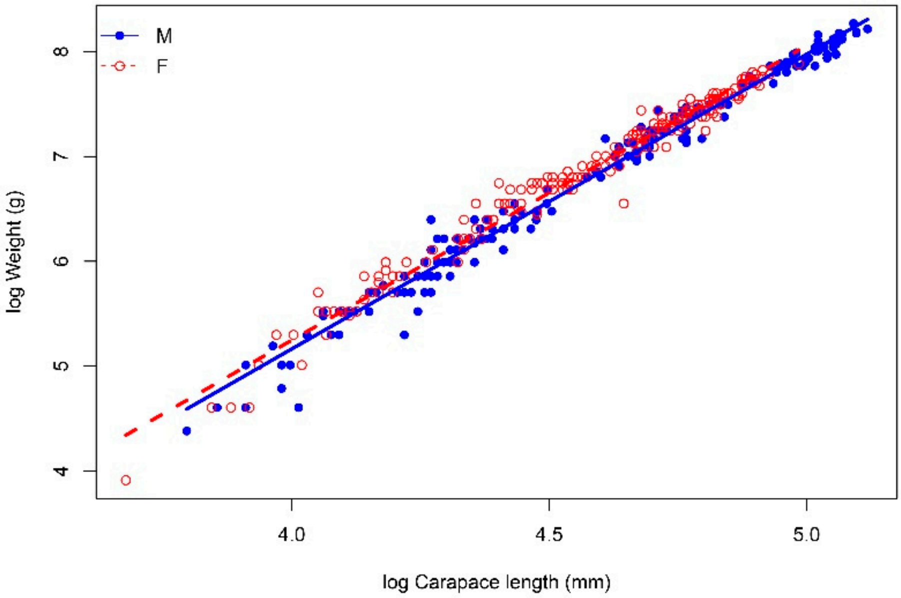

Figure S1. Plots of the log-transformed length–weight relationships illustrating observed intercept differences between males (M) and females (F) of *Palinurus elephas* from the Azores. See Table S4 for statistic results.

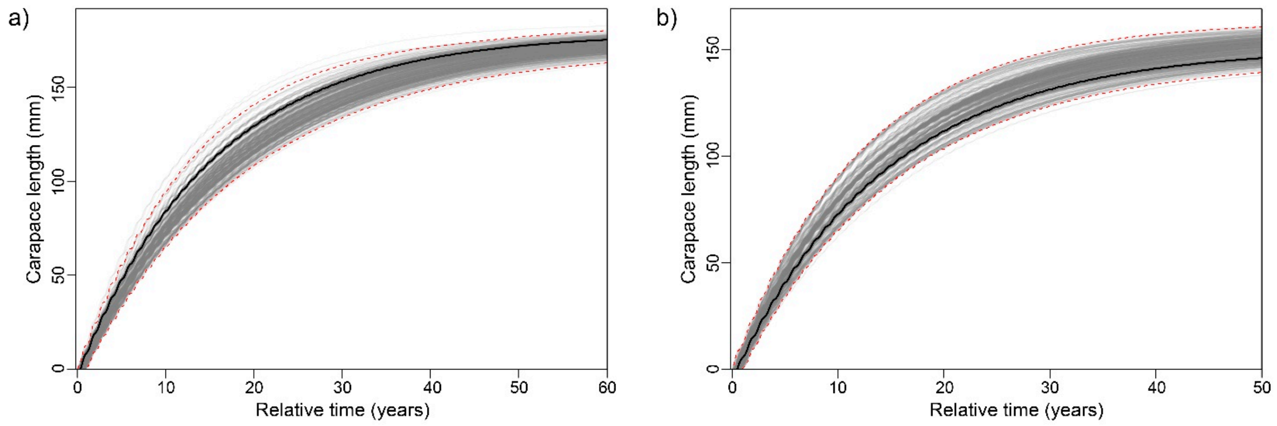

Figure S2. Von Bertalanffy growth curve and individual growth increments for males (**a**) and females (**b**) of *Palinurus elephas* from the Azores. Red dashed lines represent 0.95 confidence intervals.
